# Synthesis and anticancer activity of new flavonoid analogs and inconsistencies in assays related to proliferation and viability measurements

**DOI:** 10.3892/ijo.2014.2452

**Published:** 2014-05-21

**Authors:** ALAINA M. FORBES, HUIMIN LIN, GARY G. MEADOWS, G. PATRICK MEIER

**Affiliations:** 1 Department of Chemistry, Washington State University, Pullman, WA 99164-4630, USA; 2 Department of Pharmaceutical Sciences, Washington State University, Pullman, WA 99164-4630, USA

**Keywords:** flavonoids, quercetin, flavonol analogs, polyphenolic, polar, hydrophilic, hydrophobic, lipophilic, prostate cancer, DU-145, PC-3, human infant foreskin fibroblasts, anticancer activity, MTT, MTS, tetrazolium dye, Alamar Blue, CTG, crystal violet, Hoechst 33342, propidium iodide, rhodamine 123, trypan blue, colorimetric, cell viability, proliferation, cytotoxicity, EC_50_, therapeutic agent, Suzuki-Miyaura cross-coupling

## Abstract

Flavonoids have been studied intensely for their ability to act as anti-carcinogenic, anti-inflammatory, anti-viral and anti-aging agents and are often marketed as supplements related to their anti-inflammatory activity. Previous studies have primarily focused on the effects of polar natural flavonoids. We examined the activity of novel hydrophobic and lipophilic flavonols against human DU-145 and PC-3 prostate cancer cell lines. All flavonol analogs were more active than the naturally occurring flavonols quercetin, kaempferol, kaempferide and galangin. The most potent analogs were 6.5-fold more active against DU-145 and PC-3 cells than quercetin and fell within the biologically relevant concentration range (low micromolar). We also evaluated the potential toxic effects of flavonol analogs on normal cells, an assessment that has frequently been ignored when studying the anticancer effects of flavonoids. During these analyses, we discovered that various metabolic and DNA staining assays were unreliable methods for assessing cell viability of flavonoids. Flavonoids reduce colorimetric dyes such as MTT and Alamar Blue in the absence of cells. We showed that flavonol-treated prostate cancer cells were stained less intensely with crystal violet than untreated cells at non-toxic concentrations. The trypan blue exclusion assay was selected as a reliable alternative for measuring cell viability.

## Introduction

Numerous papers exist regarding the effects of flavonoids aiding in the prevention of cancer growth through their ability to act as anti-oxidants (1), enzyme inhibitors (2) and growth regulators (3). However, many of these papers limit their conclusions to findings on cancer cells and fail to test the impact flavonoids have on normal cells (4–7). Studies, dating back to 1977, report a connection between high flavonoid concentrations and potential mutagenic tendencies (8–13). Skibola and Smith (8), in 2000, addressed the potential danger of consuming high concentrations of flavonoids through supplements, which are not regulated by the FDA.

The flavonol, quercetin, is a common supplement promoted as having the ability to aid in cancer prevention, treat chronic infections of the prostate and other common ailments (14–16). Humans typically consume between 4 to 68 mg of flavonols daily through the consumption of fruits and vegetables (8). Flavonoid supplements, which range from 500 to 1000 mg per tablet, can provide approximately 15 times more of the flavonol than can be obtained through dietary consumption. Therefore, it is important to determine flavonoid toxicity in normal cells in order to establish if flavonoid supplements are truly safe for human consumption. Additionally, one must consider the rate of flavonoid absorption and bioavailability when designing a study to examine flavonoid activity *in vitro* (14,17–20). Flavonoids with concentrations above the absorption and bioavailability limit are not relevant to real world applications, since they will not be absorbed by the body.

Flavonoids have previously been studied as potential therapeutic agents for breast (21,22), prostate (23), lung (24,25), colon (26) and skin (27) cancers. To be a good potential therapeutic agent, flavonoids must be able to reduce cell viability in the cancerous cells, while having a minimum effect on the normal cells. In prostate cancer, polar natural flavonols (fisetin, galangin, kaempferol, morin, myricetin and quercetin) have commonly been found to exhibit these characteristics (3,28–30). However, there is limited information about the effects of hydrophobic and lipophilic flavonols on prostate cancer. The more hydrophobic (alkoxyl, geranyl > dimethylallyl > halogen > monolignol > methoxy > hydroxyl > glycosyl) and lipophilic flavonols (I > Br > Cl > F) are the most potent inhibitors of P-glycoprotein (P-gp), which is an important protein involved in drug sensitivity and resistance (31). Halogenated flavonols could also interact with Lewis bases, such as amines or alcohols of amino acids, to potentially form non-covalent halogen bonds within the cancer cells (32). Based on this limited information, a series of more hydrophobic and lipophilic analogs were synthesized with the assumption that they would decrease cell viability of DU-145 and PC-3 prostate cancer more effectively than the polar natural flavonols. The effects of flavonols on the viability of normal human infant foreskin fibroblasts (HIFF) were also studied.

**Figure 1:**
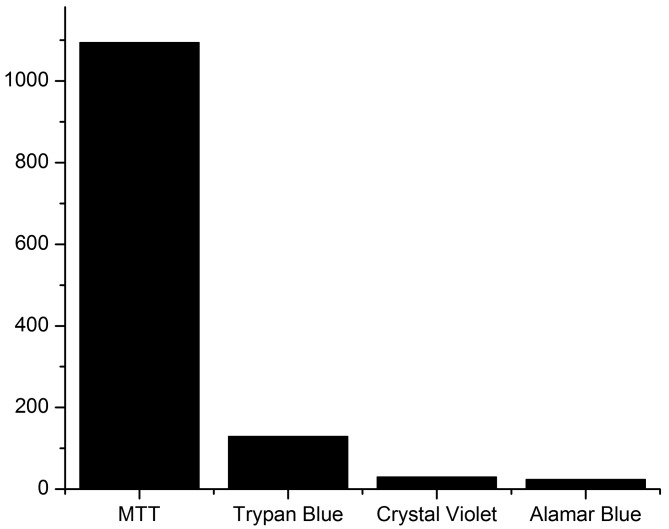
A SciFinder search found that MTT is by far the most popular cell viability detection method for flavonoid research. The SciFinder search was conducted on 01/22/14 and consisted of keywords: flavonoids; refine: MTT, trypan blue, crystal violet and Alamar Blue, respectively.

Flavonoid cell viability has most commonly been measured through colorimetric assays. However, in 2002, Bruggisser *et al* (33) found MTT to be an unreliable technique for studying flavonols due to the ability of flavonols to reduce MTT in the absence of cells. Since then, at least 1019 flavonoid articles have been published using the MTT assay. (A SciFinder search was conducted on 01/22/14 and consisted of keyword: flavonoids; refine: MTT; sort by: publication year) A recent review of the literature indicated that Alamar Blue, crystal violet and trypan blue had also been used to determine flavonoid cytotoxicity (Fig. 1). Thus, we evaluated the reliability of these lesser utilized methods in the presence of hydrophilic, hydrophobic and lipophilic flavonols to find a more accurate method of measuring flavonoid cytotoxicity.

## Materials and methods

### Chemistry reagents

All chemicals and solvents were purchased from Sigma-Aldrich (St. Louis, MO, USA). Spectra were obtained on a Perkin-Elmer Paragon 1000 FT-IR spectrometer. Proton and carbon NMR spectra were recorded on a Varian Mercury Vx 300 or 500 MHz spectrometer with (CD_3_)_2_CO and CD_3_OD as the solvents. High resolution mass spectral (HRMS) data were obtained on an Applied Biosystems/MDS SCIEX 4800 Plus MALDI TOF/TOF mass spectrometer. Melting points were determined on a Thomas Hoover Uni-melt and are uncorrected. The purity of the compounds was determined to be >95% by elemental analysis (Galbraith Laboratories, Inc., Knoxville, TN, USA).

### General procedure for the synthesis of the flavonol analogs.Synthesis of the flavonol methyl ethers

Methoxyphloroacetophenone (50 mg, 2.53 mmol) was placed into a round bottom flask, dissolved in 1,2-dichloroethane (5 ml) and 4 ml were distilled to remove water. The carboxylic acid (3.54 mmol) and the acid chloride (8.33 mmol) were then added and dissolved in triethylamine (5 ml) and dimethylformamide (DMF) (15 ml). The solution was stirred at room temperature under argon for 3 h before being heated to reflux for an additional 12 h. The triethylamine and DMF were then removed under vacuum. The residue was dissolved in 3.8 ml of ethanol and heated to reflux. A solution of KOH (1.0 g) in H_2_O (1.4 ml) was then added and the mixture was refluxed for an additional 60 min. The solvents were removed under vacuum. The solid was dissolved in water (30 ml) and the crude product was precipitated out of solution by the addition of 10% HCl until the solution reached a pH of 5.0. The crude precipitate was extracted into chloroform (5 ml) and rinsed with sodium bicarbonate (10 ml × 2). Since the desired product was only partially soluble in chloroform, these last two steps must be repeated several times (6–10 x: until the chloroform extractions are no longer yellow). The chloroform was removed under vacuum. The crude product was then dissolved in a minimal amount of acetone (~0.5 ml per 10 mg of flavonol). The solution becomes cloudy upon the addition of hexanes (10 ml) and was filtered through a pipet packed with glass wool. The filtrate contains the desired yellow product. The filtrate was evaporated under vacuum and set aside. Since some of the product remained in the precipitate, the precipitate on the glass wool in the pipet was dissolved in acetone, the acetone was evaporated, and trituration with acetone/hexanes was repeated (2–4 x: until the filtrate is no longer yellow). The NMR spectrum confirmed that the entire product was removed from the crude precipitate.

#### Deprotection

The methyl ether flavonol (20 mg, 0.055 mmol) was placed into a round bottom flask and dissolved in acetic acid (16 ml). HBr (48% aqueous solution, 2 ml) was added via a syringe. The solution was heated to reflux for 24 h. Water (150 ml) was then added, resulting in a cloudy solution. The precipitate was isolated by filtration. This crude product was dissolved in chloroform (10 ml) and rinsed with sodium bicarbonate (10 ml × 3). The chloroform was evaporated under vacuum. The crude product was dissolved in a minimal amount of acetone (0.5 ml) and an excess of hexanes (10 ml) was added. The filtrate was collected and the process was repeated several times. The filtrate was dried under vacuum to give a yellow precipitate.

##### 2-(3-Benzoylphenyl)-5,7-dihydroxy-3-methoxy-4H-chromen-4-one (Fig. 2, 4a)

55% yield (108 mg, 0.278 mmol); ^1^H NMR (300 MHz, CDCl_3_) δ 8.47 (dd, 1H, J=1.7, 3.3 Hz), 8.28 (ddd, 1H, J=1.4, 2.9, 8.1 Hz), 7.95 (ddd, 1H, J=1.4, 2.8, 7.8 Hz), 7.86 (m, 1H), 7.64 (m, 1H), 7.54 (m, 1H), 6.42 (d, 1H, J=2.0 Hz), 6.30 (d, 1H, J=2.0 Hz), 3.88 (s, 3H); ^13^C NMR (75 MHz, acetone) δ 195.7 (C), 178.3 (C), 167.8 (C), 162.1 (C), 157.4 (C), 153.8 (C), 139.6 (C), 138.0 (C), 137.4 (C), 132.9 (CH), 131.9 (CH), 131.6 (CH), 131.2 (CH), 130.00 (CH), 129.97 (CH), 129.1 (C), 128.7 (CH), 104.35 (C), 99.8 (CH), 94.4 (CH), 60.04 (CH_3_).

##### 2-(3-Benzoylphenyl)-3,5,7-trihydroxy-4H-chromen-4-one (3′-BPK) (Fig. 2, 5a)

84% yield (16 mg, 0.043 mmol); ^1^H NMR (300 MHz, acetone) δ 12.10 (s, 1H), 8.70 (dd, 1H, J=1.5, 3.0 Hz), 8.52 (ddd, 1H, J=1.3, 1.8, 8.1 Hz), 7.90 (m, 1H), 7.76 (m, 1H), 7.62 (m, 1H), 6.58 (d, 1H, J=2.1 Hz), 6.40 (d, 1H, J=1.8 Hz); ^1^H NMR (300 MHz, CDCl_3_) δ 8.47 (dd, 1H, J=1.7, 3.3 Hz), 8.28 (ddd, 1H, J=1.4, 2.9, 8.1 Hz), 7.95 (ddd, 1H, J=1.4, 2.8, 7.8 Hz), 7.86 (m, 1H), 7.64 (m, 1H), 7.54 (m, 1H), 6.42 (d, 1H), 6.30 (d, 1H); ^13^C NMR (125 MHz, MeOH) δ 197.93 (C), 177.68 (C), 166.23 (C), 162.68 (C), 158.42 (C), 145.47 (C), 139.16 (C), 138.96 (C), 138.53 (C), 134.03 (CH), 133.04 (C), 132.41 (CH), 131.92 (CH), 131.15 (CH), 130.28 (CH), 129.80 (CH), 129.62 (CH), 104.72 (C), 99.55 (CH), 94.57 (CH); Anal. Calcd. for C_22_H_14_O_6_ · H_2_O: C, 67.35%; H, 4.11%. Found: C, 66.94%; H, 4.08%.

##### 2-(4-Benzoylphenyl)-5,7-dihydroxy-3-methoxy-4H-chromen-4-one (Fig. 2, 4b)

62% yield (121 mg, 0.312 mmol); ^1^H NMR (300 MHz, acetone) δ 12.10 (s, 1H), 8.28 (ddd, 2H, J=1.8, 3.6, 8.4 Hz), 7.96 (ddd, 2H, J=1.8, 3.6, 8.7 Hz), 7.85 (m, 2H), 7.71 (dddd, 1H, J=1.7, 3.4, 7.4, 14.7 Hz), 7.60 (dddd, 2H, J=1.6, 3.2, 7.4, 14.7 Hz), 6.56 (d, 1H, J=1.8 Hz), 6.31 (d, 1H, J=1.8 Hz), 3.96 (s, 3H); ^13^C NMR (75 MHz, acetone) δ 195.09 (C), 178.84 (C), 164.77 (C), 162. 46 (C), 157.29 (C), 154.27 (C), 140.27 (C), 139.30 (C), 137.38 (C), 134.31 (C), 132.95 (CH), 129.98 (CH), 129.95 (CH), 128.72 (CH), 128.52 (CH), 105.47 (C), 99.03 (CH), 94.04 (CH), 60.14 (CH_3_).

##### 2-(4-Benzoylphenyl)-3,5,7-trihydroxy-4H-chromen-4-one (4′-BPK) (Fig. 2, 5b)

84% yield (98 mg, 0.262 mmol), dec. 221°C; IR (KBr, cm^−1^) 3283.9 (b), 1655.3 (s), 1636.8 (s), 1598.1 (s), 1501.2 (m), 1376.7 (m), 1319.5 (s), 1169.0 (s); ^1^H NMR (500 MHz, acetone) δ 12.02 (s), 8.43 (d, 2H, J=8.5 Hz), 7.95 (d, 2H, J=8.5 Hz), 7.85 (m, 2H), 7.69 (m, 2H), 7.59 (ddd, 1H, J=1.5, 7.5, 13.5 Hz), 6.61 (d, 1H, J=2.5 Hz), 6.3 (d, 1H, J=2.0 Hz); ^13^C NMR (125 MHz, acetone) δ 195.90 (C), 176.94 (C), 165.53 (C), 162.32 (C), 158.06 (C), 144.66 (C), 139.11 (C), 138.96 (C), 138.26 (C), 135.64 (C), 133.51 (CH), 130.67 (CH), 130.65 (CH), 129.36 (CH), 128.30 (CH), 104.38 (C), 99.32 (CH), 94.67 (CH); HRMS (TOF) *m/z* calcd. for C_22_H_14_O_6_ (M^+^) 375.0868, found 375.0803. Anal. Calcd for C_22_H_14_O_6_ · H_2_O: C, 67.35%; H, 4.11%. Found: C, 65.55; H, 4.10%.

##### 5,7-Dihydroxy-2-(3-iodophenyl)-3-methoxy-4H-chromen-4-one (Fig. 2, 4c)

64% yield (34 mg, 0.083 mmol); ^1^H NMR (500 MHz, MeOH) δ 8.39 (dd, 1H, J=2.0, 3.5 Hz), 8.07 (ddd, 1H, J=1.0, 1.5, 8.0 Hz), 7.90 (ddd, 1H, J=1.0, 2.0, 8.0 Hz), 7.32 (t, 1H, J=7.8, 15.5 Hz), 6.44 (d, 1H, J=2.0 Hz), 6.23, (d, 1H, J=2.0 Hz), 3.81 (s, 3H); ^13^C NMR (125 MHz, MeOH) δ 180.06 (C), 166.38 (C), 163.27 (C), 158.59 (C), 155.46 (C), 140.97 (CH), 138.05 (CH), 133.78 (C), 131.50 (CH), 128.74 (CH), 106.21 (C), 100.03 (CH), 94.90 (CH), 94.68 (C), 60.93 (CH_3_).

##### 3,5,7-Trihydroxy-2-(3-iodophenyl)-4H-chromen-4-one (Fig. 2, 5c)

90% yield (18 mg, 0.045 mmol), dec. 143°C; IR (KBr, cm^−1^) 3318.5 (b), 1690.8 (w), 1654.7 (s), 1601.9 (s), 1517.2 (m), 1375.6 (m), 1317.5 (m), 1168.3 (s); ^1^H NMR (300 MHz, acetone) δ 12.10 (s, 1H), 8.62 (dd, 1H, J=1.7, 3.3 Hz), 8.27 (ddd, 1H, J=1.2, 1.7, 8.1 Hz), 7.88 (ddd, 1H, J=1.2, 1.7, 8.1 Hz), 7.40 (t, 1H, J=8.1, 15.9 Hz), 6.59 (d, 1H, J=2.1 Hz), 6.30 (d, 1H, J=2.1 Hz); ^13^C NMR (125 MHz, MeOH) δ 177.65 (C), 166.22 (C), 162.67 (C), 158.42 (C), 144.80 (C), 139.70 (CH), 139.09 (C), 137.31 (CH), 134.71 (C), 131.27 (CH), 127.79 (CH), 104.71 (C), 99.54 (CH), 94.66 (C), 94.59 (CH); HRMS (TOF) *m/z* calcd. for C_15_H_9_IO_5_ (M^+^) 396.9573, found 396.9591. Anal. Calcd. for C_15_H_9_IO_5_ · H_2_O: C, 43.50%; H, 2.68%. Found: C, 44.39%; H 2.60%.

##### 5,7-Dihydroxy-2-(4-iodophenyl)-3-methoxy-4H-chromen-4-one (Fig. 2, 4d)

62% yield (31 mg, 0.076 mmol); ^1^H NMR (500 MHz, MeOH) δ 7.92 (ddd, 2H, J=2.1, 4.3, 8.5 Hz), 7.83 (ddd, 2H, J=2.0, 4.3, 9.0 Hz), 6.42 (d, 1H, J=2.0 Hz), 6.23 (d, 1H, J=2.5 Hz), 2.14 (s, 3H); ^13^C NMR (125 MHz, MeOH) δ 180.05 (C), 166.38 (C), 163.25 (C), 158.56 (C), 156.30 (C), 140.86 (C), 139.15 (CH), 131.32 (C), 130.98 (CH), 106.17 (C), 100.00 (CH), 98.53 (C), 94.88 (CH), 60.86 (CH_3_).

##### 3,5,7-Trihydroxy-2-(4-iodophenyl)-4H-chromen-4-one (Fig. 2, 5d)

92% yield (18.4 mg, 0.046 mmol), dec. 154°C; IR (KBr, cm^−1^) 3277.4 (b), 1686.5 (w), 1654.2 (s), 1623.6 (m), 1597.2 (s), 1499.5 (m), 1370.3 (m), 1316.8 (m), 1169.4 (s); ^1^H NMR (500 MHz, MeOH) δ 7.93 (ddd, 2H, J=2.0, 4.0, 9.0 Hz), 7.83 (ddd, 2H, J=1.5, 3.5, 8.5 Hz), 6.37 (d, 1H, J=2.0 Hz), 6.17 (d, 1H, J=2.0 Hz); ^13^C NMR (125 MHz, MeOH) δ 210.13 (C), 177.56 (C), 166.11 (C), 162.63 (C), 158.33 (C), 145.67 (C), 138.80 (CH), 132.22 (C), 130.21 (CH), 104.68 (C), 99.47 (CH), 96.85 (C), 94.55 (CH); HRMS (TOF) *m/z* calcd. for C_15_H_9_IO_5_ (M^+^) 396.9573, found 396.9592. Anal. Calcd. for C_15_H_9_IO_5_ · H_2_O: C, 43.50%; H, 2.68%. Found: C, 44.12%; H, 2.62%.

##### 5,7-Dihydroxy-3-methoxy-2-(p-tolyl)-4H-chromen-4-one (Fig. 2, 4e)

20% yield (15 mg, 0.050 mmol); ^1^H NMR (500 MHz, MeOH) δ 7.96 (d, 2H, J=8.5 Hz), 7.36 (d, 2H, J=8.0 Hz), 6.41 (d, 1H, J=2.0 Hz), 6.20 (d, 1H, J=2.0 Hz), 3.78 (s, 3H), 2.43 (s, 3H); ^13^C NMR (125 MHz, MeOH) δ 180.12 (C), 166.32 (C), 163.19 (C), 158.60 (C), 157.67 (C), 142.96 (C), 140.30 (C), 130.40 (CH), 129.40 (CH), 128.91 (C), 106.01 (C), 99.92 (CH), 94.84 (CH), 60.73 (CH_3_), 21.52 (CH_3_).

##### 3,5,7-Trihydroxy-2-(p-tolyl)-4H-chromen-4-one (Fig. 2, 5e)

80% yield (4 mg, 0.014 mmol), dec. 139°C; IR (KBr, cm^−1^) 3299.7 (b), 1700.9 (w), 1654.2 (s), 1598.1 (s), 1500.0 (m), 1371.6 (m), 1313.5 (m), 1166.6 (s); ^1^H NMR (500 MHz, acetone) δ 12.20 (s, 1H), 8.17 (d, 2H, J=8.5 Hz), 7.40 (d, 2H, J=8.0 Hz), 6.58 (d, 1H, J=2.0 Hz), 6.30 (d, 1H, J=2.0 Hz), 2.43 (s, 3H); ^13^C NMR (125 MHz, acetone) δ 176.13 (C), 164.41 (C), 161.51 (C), 157.31 (C), 145.77 (C), 140.59 (C), 136.87 (C), 129.42 (CH), 128.64 (C), 127.82 (CH), 103.60 (C), 98.47 (CH), 93.91 (CH), 20.78 (CH_3_); HRMS (TOF) *m/z* calcd. for C_16_H_12_O_5_ (M^+^) 285.0763, found 285.0783. Anal. Calcd. for C_16_H_12_O_5_ · H_2_O: C,63.57%; H, 4.67%. Found: C, 65.60%; H, 4.91%.

##### Galangin 3-methyl ether (Fig. 2, 4f)

44% yield (32 mg, 0.113 mmol); ^1^H NMR (300 MHz, acetone) δ 12.70 (s, 1H), 8.09 (m, 2H), 7.57 (m, 3H), 6.52 (d, 1H, J=2.0 Hz), 6.27 (d, 1H, J=2.0 Hz), 3.88 (s, 3H); ^13^C NMR (75 MHz, acetone) δ 178.91 (C), 164.34 (C), 162.48 (C), 157.27 (C), 155.72 (C), 139.50 (C), 131.02 (CH), 130.85 (C), 128.77 (CH), 128.52 (CH), 105.45 (C), 98.87 (CH), 93.95 (CH), 59.92 (CH_3_).

##### Galangin (Fig. 2, 5f)

92% yield (12 mg, 0.044 mmol); ^1^H NMR (500 MHz, MeOH) δ 8.19 (ddd, 2H, J=1.5, 3.0, 8.5 Hz), 7.51 (ddd, 2H, J=1.5, 7.0, 15.0 Hz), 7.45 ( ddd, 1H, J=1.5, 7.0, 16.0 Hz), 6.42 (m, 1H), 6.19 (m, 1H); ^13^C NMR (125 MHz, MeOH) δ 176.51 (C), 164.79 (C), 161.46 (C), 157.29 (C), 145.74 (C), 137.34 (C), 131.48 (C), 129.71 (CH), 128.27 (CH), 127.58 (CH), 103.50 (C), 98.21 (CH), 93.35 (CH).

### General procedure for the synthesis of aryl flavonol ethers through Suzuki-Miyaura cross-coupling reactions

The procedure developed by Molander *et al* (34) was used with minor modifications. The organotrifluoroborate (0.073 mmol), the iodo flavonol methyl ether (20 mg, 0.049 mmol), potassium carbonate (20 mg, 0.146 mmol) and Pd(OAc)_2_ (1 mg, 0.004 mmol) were placed in a round bottom flask. These compounds were dissolved in 4 ml of methanol and heated to 65°C overnight (16 h) with stirring. The solution was cooled to room temperature and 10% HCl was added until the solution reached a pH of 5.0. The resulting precipitate was isolated by filtration. The precipitate was extracted with acetone until the extracts were no longer yellow and then the acetone was removed under vacuum. The crude product was dissolved in a minimal amount of acetone (0.5 ml) and excess hexanes (10 ml) were added. The precipitate was isolated by filtration and this process was repeated several times. The filtrate was dried under vacuum to give a yellow precipitate. Deprotection of the flavonol ethers was achieved as described earlier.

#### 2-[(1,1′-Biphenyl)-3-yl]-5,7-dihydroxy-3-methoxy-4H-chromen-4-one (Fig. 3, 7c)

68% yield (30 mg, 0.083 mmol); ^1^H NMR (500 MHz, MeOH) δ 8.28 (dd, 1H, J=1.5, 3.0 Hz), 8.02 (ddd, 1H, J=1.0, 1.5, 7.5 Hz), 7.79 (ddd, 1H, J=1.0, 2.3, 7.5 Hz), 7.67 (m, 2H), 7.63 (t, 1H, J=8.0, 16.0 Hz), 7.49 (m, 2H), 7.39 (ddd, 1H, J=1.5, 3.5, 7.5 Hz), 6.43 (d, 1H, J=2.5 Hz), 6.21 (d, 1H, J=2.0 Hz), 3.83 (s, 3H); ^13^C NMR (125 MHz, MeOH) δ 180.10 (C), 163.20 (C), 158.78 (C), 157.35 (C), 143.07 (C), 141.67 (C), 140.73 (C), 137.50 (C), 132.39 (C), 130.69 (CH), 130.34 (CH), 130.10 (CH), 128.91 (CH), 128.28 (CH), 128.13 (CH), 127.99 (CH), 105.970 (C), 100.26 (CH), 95.12 (CH), 60.97 (CH_3_).

#### 2-[(1,1′-Biphenyl)-3-yl]-3,5,7-trihydroxy-4H-chromen-4-one (Fig. 3, 8c)

93% yield (18 mg, 0.051 mmol), dec. 138°C; IR (KBr, cm^−1^) 3331.1 (b), 1686.1 (w), 1654.4 (s), 1603.6 (s), 1508.3 (m), 1375.4 (m), 1319.9 (m), 1169.0 (s); ^1^H NMR (500 MHz, acetone) δ 8.54 (s, 1H), 8.25 (d, 1H, J=8.0 Hz), 7.80 (d, 1H, J=7.5 Hz), 7.75 (d, 2H, J=7.5 Hz), 7.68 (t, 1H, J=7.5, 15.0 Hz), 7.53 (m, 2H), 7.43 (t, 2H, J=7.5, 15.0 Hz), 6.61 (d, 1H, J=2.0 Hz), 6.30 (d, 1H, J=2.0 Hz); ^13^C NMR (125 MHz, acetone) δ 176.32 (C), 164.75 (C), 161.78 (C), 157.41 (C), 145.41 (C), 141.61 (C), 140.74 (C), 132.09 (C), 129.39 (CH), 129.22 (CH), 128.77 (CH), 127.95 (CH), 127.28 (CH), 126.81 (CH), 126.38 (CH), 103.75 (C), 98.66 (CH), 94.08 (CH); HRMS (TOF) *m/z* calcd. for C_21_H_14_O_5_ (M^+^) 347.0919, found 347.0967. Anal. Calcd. for C_21_H_14_O_5_ · H_2_O: C, 69.23%; H, 4.43%. Found: C, 69.09%; H, 4.30%.

#### 2-[(1,1′-Biphenyl)-4-yl]-5,7-dihydroxy-3-methoxy-4H-chromen-4-one (Fig. 3, 7d)

67% yield (6 mg, 0.017 mmol); ^1^H NMR (500 MHz, acetone) δ 12.70 (s, 1H), 8.21 (ddd, 2H, J=2.0, 3.9, 4.0, 8.5 Hz), 7.87 (ddd, 2H, J=2.0, 3.0, 9.0 Hz), 7.77 (m, 2H), 7.52 (m, 2H), 7.43 (dddd, 1H, J=1.5, 2.5, 7.5, 15.0 Hz), 6.55 (d, 1H, J=2.0 Hz), 6.28 (d, 1H, J=2.0 Hz), 3.93 (s, 3H); ^13^C NMR (125 MHz, acetone) δ 179.62 (C), 165.04 (C), 163.25 (C), 158.01 (C), 156.04 (C), 144.07 (C), 140.60 (C), 130.44 (C), 129.90 (CH), 129.77 (CH), 128.97 (CH), 127.84 (CH), 127.83 (CH), 106.11 (C), 99.47 (CH), 94.56 (CH), 60.49 (CH_3_).

#### 2-[(1,1′-Biphenyl)-4-yl]-3,5,7-trihydroxy-4H-chromen-4-one (Fig. 3, 8d)

98% yield (6 mg, 0.016 mmol), dec. 148°C; IR (KBr, cm^−1^) 3299.7 (b), 1702.1 (w), 1654.3 (s), 1625.1 (s), 1603.4 (s), 1508.0 (m), 1384.0 (m), 1314.4 (m), 1166.2 (s); ^1^H NMR (500 MHz, acetone) δ 12.10 (s, 1H), 8.36 (ddd, 2H, J=2.0, 4.3, 8.5 Hz), 7.87 (ddd, 2H, J=2.0, 3.5, 9.0 Hz), 7.77 (m, 2H), 7.51 (dddd, 2H, J=1.5, 4.0, 8.0, 15.0 Hz), 7.41 (dddd, 1H, J=1.3, 2.6, 7.5, 15.0 Hz), 6.59 (d, 1H, J=2.0 Hz), 6.29 (d, 1H, J=2.0 Hz); ^13^C NMR (125 MHz, acetone) δ 176.82 (C), 165.26 (C), 162.41 (C), 157.99 (C), 145.87 (C), 143.15 (C), 140.76 (C), 138.05 (C), 131.04 (C), 129.86 (CH), 129.01 (CH), 128.82 (CH), 127.79 (CH), 127.76 (CH), 104.33 (C), 99.24 (CH), 94.61 (CH); HRMS (TOF) *m/z* calcd. for C_21_H_14_O_5_ (M^+^) 347.0919, found 347.0995. Anal. Calcd. for C_21_H_14_O_5_ · H_2_O: C, 69.23%; H, 4.43%. Found: C, 68.21%; H, 4.27%.

### Biological reagents

Quercetin, kaempferide, kaempferol and morin were purchased from Indofine (Hillsborough, NJ, USA). Vi-cell reagent Pak was purchased from Beckman Coulter (Fullerton, CA, USA). Alamar Blue was purchased from BioSource International (Camarillo, CA, USA).

### Culture of prostate cancer and HIFF cells

DU-145 and PC-3 were obtained from the American Type Tissue Collection (Rockville, MD, USA). HIFF were a gift from Dr Mary Sanchez-Lanier, Department of Microbiology, Washington State University. HIFF are a fibroblast cell line that was isolated from human infant foreskin under written consent from the minor’s parent (approval #60277 from the Washington State University Institutional Review Board). HIFF and the DU-145 cells were cultured in DMEM (Hyclone, Logan, UT, USA) containing 1% penicillin/streptomycin (Mediatech Incorporated, Herndon, VA, USA) and 10% FBS (Equitech-Bio Inc., Kerrville, TX, USA). The PC-3 cells were cultured in RPMI-1640 (Invitrogen Corp., Carlsbad, CA, USA) containing 10% FBS and 1% penicillin/streptomycin. All of these cells were seeded into 24-well plates and kept in a 37°C humidified incubator with 5% CO_2_.

### Treatment and measurement of cell viability

Stock solutions of the natural product and synthetic flavonol analogs were prepared by dissolving them in DMSO. Preliminary dose-response studies were conducted in the prostate cancer and HIFF cell lines with each flavonol to find the minimum and maximum effective dose range and an approximate EC_50_. The actual EC_50_ for each flavonol was calculated from a minimum of 6 concentrations selected within the linear portion of initial dose range curves and bracketing the estimated EC_50_. Cells were seeded into 24-well plates 24 h prior to the experiment at the following cell densities: DU-145 (6,000 cells/well), PC-3 (6,500 cells/well) and HIFF (6,000 cells/well). The stock solutions of the flavonols were added to each of the wells, except the control, to give the desired concentration of the flavonols and a final concentration of 0.2% DMSO and the plates were incubated for 72 h. DMSO did not alter the growth or viability of DU-145, PC-3 or HIFF (data not shown). Assays were run in quadruplicate. The reliability of three cell viability assays was tested with the following reagents: i) Alamar Blue: After removal of the culture medium, cells were washed with PBS and incubated with Alamar Blue for 6 h. Fluorescence was measured with an excitation at 530 nm and an emission at 590 nm. ii) Crystal violet: After removal of the culture medium, the cells were fixed with 200 μl of 4% formaldehyde at 4°C for 30 min, washed with a 3:1 mixture of MeOH: AcOH, rinsed with 80% MeOH, and stained with 0.1% crystal violet. After 1 h at room temperature, the wells were gently rinsed with distilled water, dried, dissolved in 10% AcOH and the absorbance was read at 570 nm. iii) Trypan blue exclusion: The cells were trypsinized, washed with calcium free phosphate buffered saline (PBS), centrifuged and resuspended in 500 μl of PBS. Cell viability was accurately determined using the semi-automated Vi-Cell XR Cell Analyzer (Beckman Coulter).

### Statistical analysis

The EC_50_ values of the flavonols in each cell line were analyzed by one-way analysis of variance (ANOVA). Comparisons of the EC_50_ values of the flavonols between cell lines were evaluated by two-way ANOVA. Differences were considered significant at P<0.05.

## Results

### Chemistry

**Figure 2:**
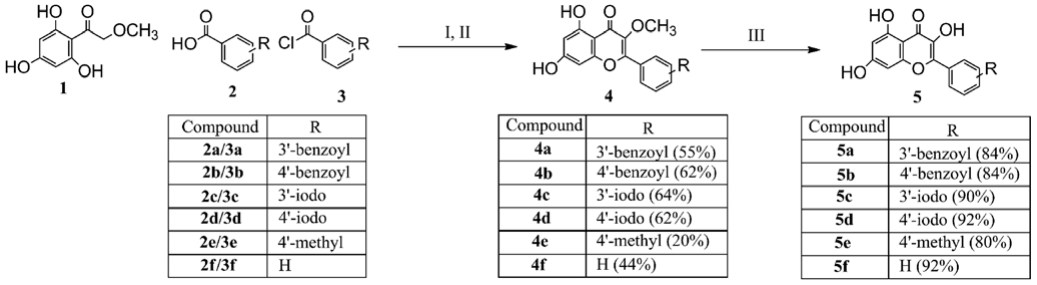
Synthesis of flavonol analogs. Reagents and conditions: (I) Et_3_N, DMF, 2, 3, reflux (II) KOH/H_2_O, ethanol, reflux (III) HBr, acetic acid, reflux.

A series of flavonol analogs were synthesized from the starting material, ω-methoxyphloroacetophenone 1 (Fig. 2) (23,35,36). Initially, these compounds were synthesized through a modified multistep procedure by Tanaka *et al* (37), in which the ω-methoxyphloroacetophenone 1 was esterified by the acid chloride 3 to produce a triester intermediate, which was subsequently cyclized by refluxing in pyridine containing K_2_CO_3_ to give the flavonol methyl ether 4. This sequence resulted in moderate net yields of the flavonol methyl ethers. A major reason for the modest yields was the poor recovery of the products from the chromatographic purifications at each step. Therefore, we explored an alternative approach, the ‘one pot’ procedure described by Ichikawa *et al* (38) followed by ester hydrolysis, to synthesizing the flavonol methyl ether 4. Ichikawa *et al* used Et_3_N as both the solvent and the base. This procedure resulted in slightly higher yields (26–35%) of the flavonol methyl ether 4 than the previous process. During these reactions we noted the low solubility of the ω-methoxyphloroacetophenone in Et_3_N. Based on these observations, the yields of compound 4 were increased through a series of optimization reactions (solvent, temperature and reaction time). These studies led to the use of Et_3_N as the base and DMF as the solvent to give a homogenous solution, which significantly increased the yields of the cyclized products. With these optimal conditions, good yields were achieved in the synthesis of the hydrophobic aromatic and halogenated flavonol ethers 4a–d [3′-benzoyl (55%) and 4′-benzoyl (62%), 3′-iodo (64%) and 4′-iodo (62%)]. Moderate to low yields were observed in the synthesis of the smaller, less hydrophobic and non-substituted flavonol ethers 4e–f [H-flavonol (44%) and 4′-methyl (20%)]. Demethylation of the methyl ethers of flavonols was known to occur under strong, anhydrous Lewis Acid conditions (i.e. AlBr_3_, CH_3_CN) (39), so we were pleasantly surprised to find that deprotection of 4 could be achieved under the relatively milder conditions of aqueous HBr in acetic acid (40), to form 5a–f in excellent yields [3′-benzoyl (37) (84%) and 4′-benzoyl (84%), 3′-iodo (90%) and 4′-iodo (41) (92%), H-flavonol (42–45) (92%) and 4′-methyl (80%) flavonols].

**Figure 3:**
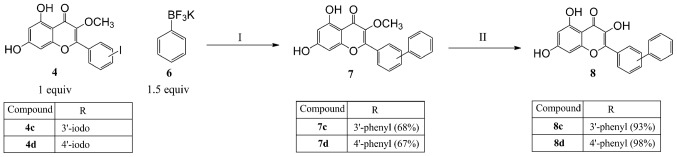
Synthesis of biaryl flavonols. Reagents and conditions: (I) Pd(OAc)_2_, K_2_CO_3_ (3 equiv), MeOH, 65°C (II) HBr, acetic acid, reflux.

The remaining two analogs (3′- and 4′-phenyl) were synthesized through a Suzuki-Miyaura palladium catalyzed cross-coupling reaction using a procedure developed by Molander *et al* (34) (Fig. 3). The 3′- and 4′-iodo flavonol ethers 4c,d readily coupled with potassium phenyl trifluoroborate using Pd(OAc)_2_ and potassium carbonate in methanol to give high yields of both 3′-phenyl 7c (68%) and the 4′-phenyl 7d (67%) flavonol ethers. These compounds were readily demethylated to the 3′-phenyl 8c (93%) and 4′-phenyl 8d (98%) flavonols using the previously determined reaction conditions.

### Biological method validation of cell viability assays

**Figure 4:**
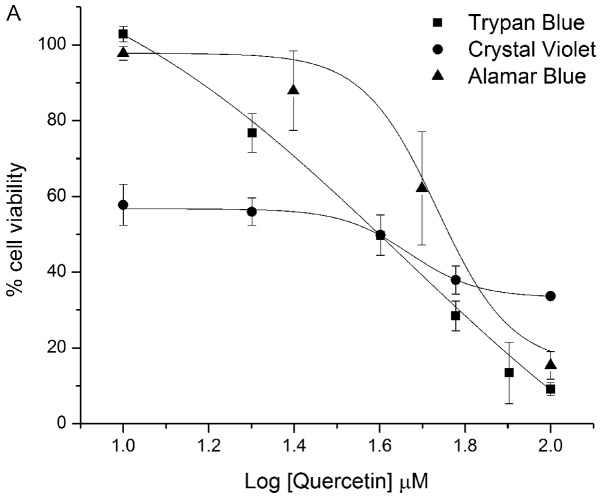

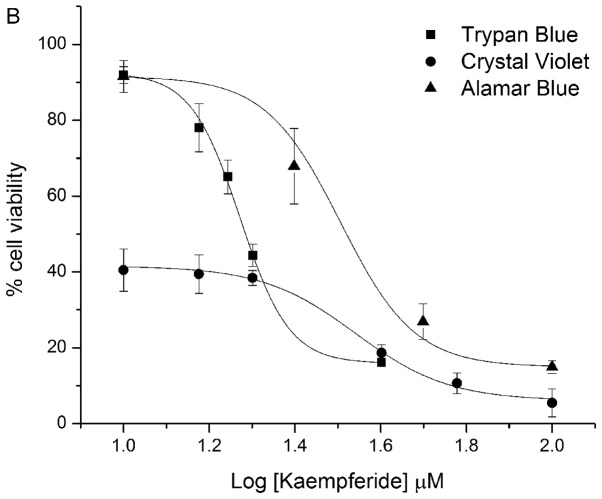
The effect of quercetin (A) and kaempferide (B) on cell viability in prostate cancer cells, DU-145. Values are mean ± SEM of 3 or more independent experiments.

The reliability of various colorimetric and cell counting assays in the presence of flavonols was determined by comparing the metabolism-dependent Alamar Blue assay, the DNA-binding crystal violet assay and the dye exclusion trypan blue method. We found that flavonols reduced Alamar Blue in the absence of cells and overestimated the number of viable prostate cancer cells (Fig. 4). Flavonols also interfered with the crystal violet assay by staining untreated cells more intensely than flavonol treated cells. For many flavonol treated cells, the maximum achievable cell viability was only 50% compared to the control, even at non-cytotoxic concentrations. For some flavonols, such as kaempferide, the maximum cell viability did not ever reach 50%.

**Figure 5:**
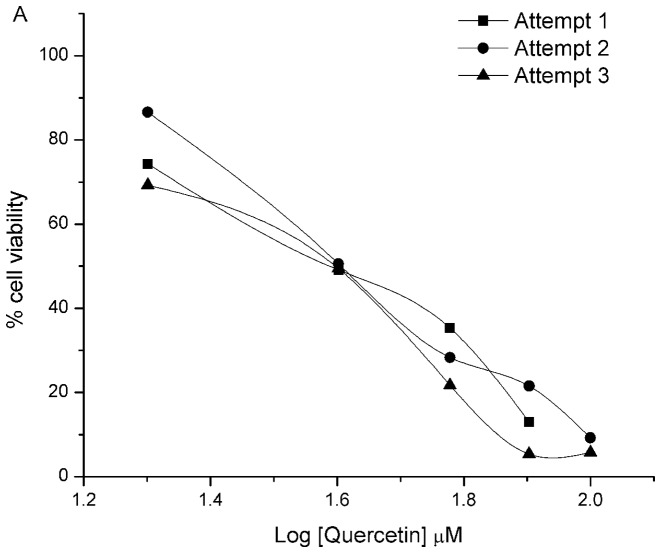

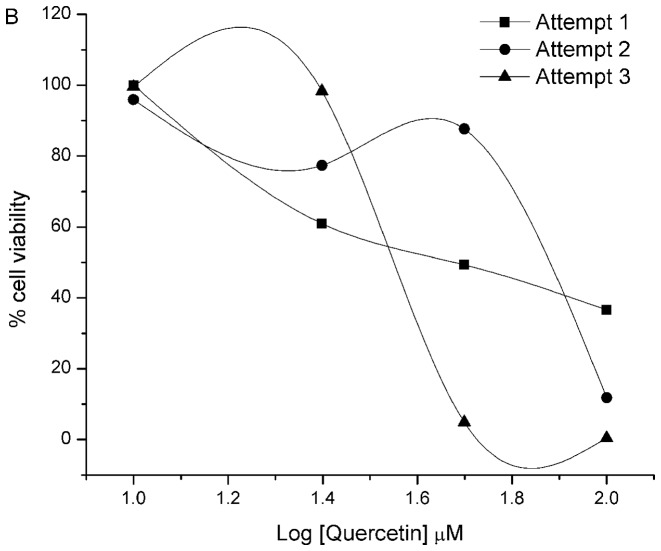
Precision of three individual assays for quercetin cytotoxicity against prostate cancer cells, DU-145; Trypan blue (A) and Alamar Blue (B).

The trypan blue cell counting assay proved to be the most reliable method for measuring cell viability in the presence of flavonols with a minimal difference (≤10%) between the total number of cells counted and the number of cells stained with trypan blue. These results indicated that flavonols did not interfere with the cell counting readings and provided a model by which to compare the reliability of other assays such as Alamar Blue and crystal violet. A comparison between trypan blue and Alamar Blue cell viability curves showed a shift in the Alamar Blue curve to the right, which is in agreement with previous studies that phenolic compounds resulted in an overestimation of cell viability (Fig. 4). Quercetin had an EC_50_ value of 55.1 μM in Alamar Blue compared to 39.1 μM in trypan blue. Kaempferide had an EC_50_ value of 30.1 μM in Alamar Blue compared to 19.8 μM in trypan blue. We also found, for some compounds, that the reproducibility of Alamar Blue was poor (Fig. 5). This may have resulted from inconsistent flavonol reduction of the metabolic compound between analyses with fluctuating variables, such as the amount of flavonols remaining in the well during incubation and cell numbers between analyses. The major finding using the crystal violet assays was that flavonols significantly masked the effects on cell viability readings at lower concentrations (Fig. 4). However, at higher flavonol concentrations this interference appeared to be minimal and comparable to the trypan blue assay.

### Effects of flavonols on the in vitro viability of DU-145 and PC-3 prostate cancer cells

Flavonols with a carbonyl at C4, a double bond between C2 and C3, and a hydroxyl group at position 3 of ring C, were selected for this study to determine cytotoxicity towards androgen-independent DU-145 and PC-3 human prostate cancer cells in comparison to toxicity towards HIFF normal cells as determined using the trypan blue exclusion assay. All of the flavonols tested decreased the number of viable DU-145 and PC-3 cells. In DU-145 cells, the more hydrophobic and lipophilic flavonol analogs (3′- and 4′-phenyl and 4′-iodo flavonols) were the most potent (Table I). The other hydrophobic and lipophilic flavonols (3′-iodo, 3′- and 4′-benzoyl and 4′-methyl flavonols) as well as the natural product, kaempferide, were slightly less efficient at decreasing cell viability. The natural product, galangin, moderately decreased viability, while quercetin and kaempferol had the least effect.

In PC-3 cells, the more hydrophobic and lipophilic analogs, 4′-iodo and 4′-phenyl flavonols, were the most effective compounds at reducing cell viability (Table I). Other hydrophobic compounds (3′-phenyl, 3′- and 4′-benzoyl, 4′-methyl flavonols, kaempferide and galangin) were slightly less active, exhibiting EC_50_ values similar to each other. The least hydrophobic flavonols, kaempferol and quercetin, were clearly identified as the least efficacious compounds.

**Figure 6:**
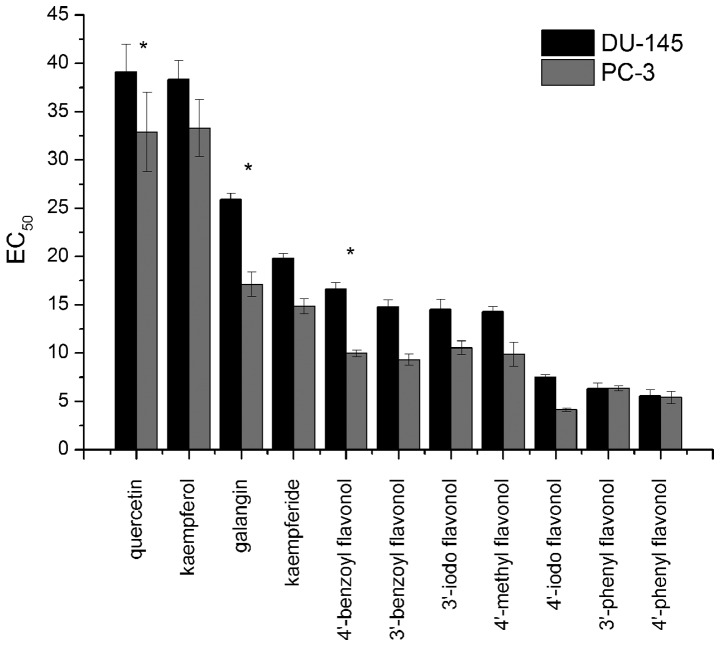
Two-way ANOVA comparison of the differences between the EC_50_ values of the two prostate cell lines, DU-145 and PC-3. The EC_50_ values for quercetin, galangin and 4′-benzoyl flavonol were statistically different between the two cell lines (^*^P<0.05). Values are mean ± SEM.

A comparison of the differences in the EC_50_ values of the flavonols between the two prostate cancer cell lines showed that only three compounds, quercetin, galangin and 4′-benzoyl flavonol, exhibited differential cytotoxicity (Fig. 6). All three of these compounds were found to have a greater effect on viability of PC-3 than on DU-145 cells (P<0.05). Of the flavonols tested, 4′-iodo, 3′-phenyl and 4′-phenyl flavonol proved to have the greatest effect on cell viability, reducing the number of viable prostate cells by 6.5-fold compared to the natural product, quercetin.

The EC_100_ values for DU-145 and PC-3 showed a similar trend to their EC_50_ values (Table II). The more hydrophobic and lipophilic analogs (3′- and 4′-phenyl, 3′- and 4′-iodo, 3′-benzoyl and 4′-methyl flavonols) had the lowest EC_100_ values. The natural products, kaempferide and galangin, were slightly less efficient at achieving their maximum effect. Quercetin and kaempferol were the least effective. A comparison of the differences in EC_100_ values of flavonols between these two cell lines showed that only two compounds, kaempferol and 4′-benzoyl, were statistically different (data not shown). Both compounds were more effective against PC-3 than DU-145 cells (P<0.05). From these results, we concluded that only a minimum concentration (<25 μM) of the more hydrophobic and lipophilic flavonol analogs (3′- and 4′-phenyl, 3′- and 4′-iodo, 3′-benzoyl and 4′-methyl flavonols) was needed to achieve a maximum effect on both the DU-145 and PC-3 cell lines.

A comparison of the difference between the EC_100_ and EC_50_ value of the flavonols in DU-145 showed that only a small increase in concentration (10 μM or less) was needed to achieve maximal effect for the hydrophobic and lipophilic analogs (4′-phenyl, 3′-benzoyl, 4′-methyl, 3′- and 4′-iodo flavonols) (Table III). The natural products, galangin and kaempferide, as well as the flavonol analog, 4′-methyl flavonol, had a slightly larger range (<25 μM) with 3′-phenyl flavonol overlapping the two categories. Quercetin and kaempferol were the least effective and the range between the EC_100_ and EC_50_ values was at least a 36 μM.

In PC-3 cells, a 10 μM or less difference between the EC_100_ and EC_50_ values was found in the more hydrophobic and lipophilic flavonol analogs (4′-phenyl, 3′-benzoyl, 3′-phenyl, 3′- and 4′-iodo flavonols) (Table III). The less hydrophobic natural products, kaempferol, galangin and kaempferide, required a slightly larger range (<25 μM) to achieve a maximal effect with the 4′-methyl and 4′-benzoyl flavonol overlapping the two categories. Quercetin was the least effective and the range between the EC_100_ and EC_50_ values was 44 μM.

A comparison of the differences in the EC_100_ and EC_50_ values of flavonols between these two cell lines showed that only two compounds, kaempferol and 4′-benzoyl flavonol, were statistically different (data not shown). These results indicate that hydrophobic and lipophilic flavonol analogs tend to reach their maximal effect at lower concentrations than natural flavonols and that this maximal effect is achieved over a more narrow effective concentration range (with 4′-benzoyl flavonol being the exception in DU-145). Narrow ranges between the EC_50_ and EC_100_ values are beneficial because they reduce the likelihood that the agents will be toxic to normal cells.

### Effects of flavonols on the in vitro viability of HIFF normal cells

HIFF cells were used to determine the toxicity of flavonols on normal cells. Flavonoid research has commonly been conducted at concentrations of 50 μM or greater (5,46). Based on this information, we first examined the effects of flavonols on HIFF cells at 50 μM concentrations. At this concentration, we found that all flavonols tested caused a decrease in cell viability (Table IVA). Quercetin was the least inhibitory and all of the synthesized derivatives exhibited major inhibitory activity at the 50 μM concentration.

HIFF cell viability was also examined at the average flavonol EC_50_ concentrations (for DU-145 and PC-3) for individual compounds. At this concentration prostate cancer cells would have a cell viability of 50%. Kaempferol, 4′-benzoyl and 3′-iodo flavonols had no affect on the cell viability of HIFF cells (Table IVB). The natural products, quercetin and galangin, and the flavonol analog, 3′-benzoyl flavonol, caused a slight decrease in the viability of HIFF cells. A moderate effect was observed for kaempferide and 4′-phenyl flavonol. Three flavonols, 4′-methyl, 4′-iodo and 3′-phenyl flavonol exhibited the strongest decrease in cell viability. The activity of flavonols towards HIFF cells did not correlate with the activity of flavonols towards the prostate tumor cells. A comparison of the flavonols, which minimally decreased the viability of HIFF cells and efficiently decreased the viability in prostate cancer cells at both the EC_50_ and EC_100_ values, lead us to conclude that 3′-iodo and 3′-benzoyl flavonols are likely to be the most promising therapeutic agents.

## Discussion

For decades, flavonoids have been extensively studied for their ability to act as anti-oxidants, enzyme inhibitors and growth regulators. More recently, flavonols have become available as supplements. In preparing to examine the effects of new flavonol analogs on cell viability of human prostate cancer cells, we found that many previous reports failed to use reliable methods to examine this effect and in addition, a number of studies examined the effects of non-biologically relevant concentrations of flavonoids on cell viability. Cell viability and proliferation (47–50) are commonly measured through metabolic [MTT, MTS, Alamar Blue and CTG (Cell-Titer Glo)], DNA or protein-binding (crystal violet, Hoechst 33342, propidium iodide and rhodamine 123) and cell-based (trypan blue) assays.

Metabolic assays measure the metabolism of the colorimetric, fluorescent and/or luminescent compound in live cells. MTT (51), MTS (52) and Alamar Blue (53) are reduced by NAD(P)H-dependent oxidoreductase enzymes, while CTG (54) is reduced by cellular ATP. Several of these agents have previously proven unreliable in the presence of flavonoids. For example, flavonoids reduce MTT (33,55,56) and MTS (57), a second generation tetrazolium dye, in the absence of cells and CTG (22) overestimates the number of viable cells for some polyphenolic compounds. In this study, we found that flavonoids also reduce Alamar Blue in the absence of cells and overestimate the number of viable cells. For NAD(P)H-dependent assays this interference is likely due to the ability of flavonoids to mimic the reducing agent, NAD(P)H. Flavonoids are known anti-oxidants, capable of preventing oxidative damage by being oxidized themselves (58–60). Several anti-oxidants (flavonoids, ascorbic acid, vitamin E, ubiquinone, hydroquinone and N-acetylcysteine) also reduce MTT in the absence of cells suggesting that MTT and other metabolic-dependent agents are not reliable assays for analyzing agents that have anti-oxidant activity (33,61–64). For ATP-dependent assays, flavonoids likely mimic ATP by binding to the ATP binding site (2,29,31). Several flavonols, such as quercetin, kaempferol and galangin are known to exhibit this property.

Flavonoids also interfere with the staining of DNA (Hoechst 33342, propidium iodide and crystal violet) and proteins (rhodamine 123). For example, the flavonol, quercetin, previously was found to inhibit the binding of Hoechst 33342 to DNA and to activate the binding of rhodamine 123 to proteins (31,65). The anthocyanin, cyanidin-3-rutinoside, prevented the binding of propidium iodide to DNA (66). These dyes have commonly been used in flow cytometry studies. We found that crystal violet stained the DNA of untreated cells more intensely than flavonol treated cells. In several cases, the maximum achievable cell viability was only 50% of the control at nontoxic concentrations. The interference of the flavonoids with the ability of crystal violet to bind the DNA might have resulted from intercalation of the flavonols into DNA, since flavonols such as quercetin and kaempferol are known to exhibit this property (67). Rhodamine 123 measures mitochondrial membrane potential, which is regulated by ATP (68). Therefore, if quercetin binds to the ATP binding site it would disrupt the membrane potential and would interfere with the accumulation of rhodamine 123 inside the membrane. Additionally, quercetin enhances the rate of rhodamine 123 transport into P-glycoproteins (69).

In order for these assays to be reliable, excess flavonoids must be removed before the addition of the metabolic agent. Previous investigators have suggested removing the culture medium before the addition of the metabolic agent (33). However, flavonoids are minimally soluble in biological buffers. Therefore, this method is inefficient at removing all of the flavonoids in solution and in addition, does not account for the flavonoids located within the cell that can also interfere with the colorimetric agent. Liu *et al* (70) previously found in B12 cells, which are phenotypically similar to neural precursor cells, that genistein reduced 60% more MTT than the control during a 3 h incubation. Therefore, the impact that flavonoids have on metabolic agents may vary depending on the amount and type of flavonoid used.

Based on these results, we concluded that, though popular, metabolic and DNA staining assays are not a reliable method of measuring cell viability and proliferation in the presence of flavonoids. The trypan blue cell counting assay provides an alternative method to these assays, but has its own set of limitations (71–73). Some color variations may be undetectable to the human eye and debris can also be stained and counted as a nonviable cell. For accurate results, early phase cells (≤72 h) must be used to prevent an overestimation of the number of viable cells and cells must be counted within 5 min of the addition of the dye. An automatic cell viability analyzer such as the Vi-Cell XR (Beckman Coulter Inc.) addresses most of these limitations by more accurately detecting different sizes and shades of stained cells, allowing rapid analysis (<3 min) and providing more consistent results than the manual method (74). Additionally, the difference between the total number of cells and the number of nonviable cells was ≤10%, implying that flavonols did not interfere with the cell counting readings. Our results correlate well with previously published trypan blue results. For example, Vijayababu *et al* (75) found in PC-3 cells that quercetin had an EC_50_ value of around 29 μM compared to our 32.9 μM.

Polar natural flavonols (quercetin, kaempferol, kaempferide and galangin) have commonly been the focus of flavonoid cancer research. However, their *in vitro* anticancer effects have not translated well to their effects *in vivo*, probably, because absorption and bioavailability of dietary flavonoids (4–68 mg) are limited to the low micromolar range (76–78) and previously, their effective and cytotoxic dosages have been well above this concentration limit. For example, the flavonol supplement quercetin has an EC_50_ value of 39.1 μM in DU-145 and 32.9 μM in PC-3 cells. We found that increasing the hydrophobicity and lipophilicity of a flavonol significantly lowers the effective concentration of several analogs (4′-iodo, 3′-phenyl and 4′-phenyl flavonol) into the desired low micromolar range thus enhancing their therapeutic potential. However, information about the absorption and bioavailability of these novel flavonols are needed to provide a more complete picture of their ultimate anticancer potential *in vivo*.

## Figures and Tables

**Table I:** EC_50_ of DU-145 and PC-3.

Flavonol	EC_50_ values in DU-145 cells	Flavonol	EC_50_ values in PC-3 cells
Quercetin	39.11±2.87^a^	Quercetin	32.87±4.10^e^
Kaempferol	38.35±1.94^a^	Kaempferol	33.29±2.96^e^
Galangin (H-flavonol 5f)	25.94±0.59^b^	Galangin (H-flavonol 5f)	17.11±1.29^f^
Kaempferide	19.82±0.50^c^	Kaempferide	14.88±0.78^g^
4′-Benzoyl flavonol (5b)	16.61±0.70^c^	3′-Iodo flavonol (5c)	10.54±0.72^h^
3′-Benzoyl flavonol (5a)	14.81±0.71^c^	4′-Benzoyl flavonol (5b)	9.97±0.36^h^
3′-Iodo flavonol (5c)	14.53±1.02^c^	4′-Methyl flavonol (5e)	9.89±1.24^h^
4′-Methyl flavonol (5e)	14.30±0.53^c^	3′-Benzoyl flavonol (5a)	9.31±0.58^h^
4′-Iodo flavonol (5d)	7.51±0.27^d^	3′-Phenyl flavonol (8c)	6.35±0.26^i^
3′-Phenyl flavonol (8c)	6.33±0.58^d^	4′-Phenyl flavonol (8d)	5.40±0.63^j^
4′-Phenyl flavonol (8d)	5.57±0.60^d^	4′-Iodo flavonol (5d)	4.13±0.16^k^

EC_50_ values (μM) are mean ± SEM of 3 independent experiments except for 4′-methyl flavonol in PC-3 cells in which the mean reflects 4 independent experiments. For all flavonols P<0.05. Tukey’s multiple comparison tests determined statistical differences between the EC_50_ values for each flavonol after ANOVA. *DU-145:* a, Quercetin and kaempferol are different from groups b, c and d, but not from each other. b, Galangin is different from all other compounds. c, These flavonols are different from groups a, b and d, but not from each other. d, The 4′-iodo and the 3′- and 4′-phenyl flavonols are different than group a, b and c, but not from each other. *PC-3:* e, Quercetin and kaempferol are different from groups f, g, h, i, j and k, but not from each other. f, Galangin is different from groups e, j and k as well as 4′-methyl flavonol, but not from kaempferide, 3′-iodo and 4′-benzoyl flavonols. g, Kaempferide is different from group e, j and k, but not galangin, 3′-iodo, 4′-benzoyl and 4′-methyl flavonols. h, 3′- and 4′-benzoyl, 4′-methyl and 3′-iodo flavonols are the same as all flavonols except for quercetin, kaempferol and galangin. i, 3′-phenyl flavonol is the same as all flavonols except for group e and galangin and kaempferide. j, 4′-phenyl flavonol is different from all flavonols except for 3′-benzoyl, 3′-phenyl and 4′-iodo flavonols. k, 4′-iodo flavonol is different from all flavonols except 3′- and 4′-phenyl flavonols.

**Table II:** EC_100_ values of DU-145 and PC-3.

Flavonol	EC_100_ values in DU-145 cells	Flavonol	EC_100_ values in PC-3 cells
Quercetin	85.42±6.11^a^	Quercetin	77.13±3.40^f^
Kaempferol	74.36±0.83^a^	Kaempferol	57.62±6.40^g^
Galangin (H-flavonol 5f)	46.42±3.92^b^	Galangin (H-flavonol 5f)	43.92±4.14^h^
4′-Benzoyl flavonol (5b)	41.04±2.18^b^	Kaempferide	34.16±1.66^h^
Kaempferide	39.19±0.87^b^	4′-Benzoyl flavonol (5b)	22.31±0.91^i^
3′-Iodo flavonol (5c)	24.61±2.06^c^	4′-Methyl flavonol (5e)	20.38±1.77^j^
4′-Methyl flavonol (5e)	23.75±2.53^c^	3′-Iodo flavonol (5c)	19.54±1.08^k^
3′-Benzoyl flavonol (5a)	20.43±0.41^d^	3′-Benzoyl flavonol (5a)	17.86±1.56^k^
3′-Phenyl flavonol (8c)	18.00±0.61^d^	3′-Phenyl flavonol (8c)	11.33±1.38^k^
4′-Phenyl flavonol (8d)	12.43±1.85^d^	4′-Iodo flavonol (5d)	10.34±0.73^l^
4′-Iodo flavonol (5d)	9.84±0.33^e^	4′-Phenyl flavonol (8d)	8.75±0.45^m^

EC_100_ values (μM) are mean ± SEM of 3 independent experiments except for 4′-methyl flavonol in PC-3 cells in which the mean reflects 4 independent experiments. For all flavonols P<0.05. Tukey’s multiple comparison tests determined statistical differences between the EC_100_ values for each flavonol after ANOVA. *DU-145*: a, Quercetin and kaempferol are different from groups b, c, d and e, but not from each other. b, Galangin, kaempferide and 4′-benzoyl flavonol are different from groups a, c, d and e, but not from each other. c, The 3′-iodo and 4′-methyl flavonol are different from all flavonols except 3′-benozyl, 3′-phenyl and 4′-phenyl flavonol as well as each other. d, The 3′-benzoyl, 3′-phenyl and 4′-phenyl flavonol are the same as all flavonols except for groups a and b. e, The 4′-iodo flavonol is different from all flavonols except 3′-benzoyl, 3′-phenyl and 4′-phenyl flavonol. *PC-3*: f, Quercetin is different from all other compounds. g, Kaempferol is different from all other compounds. h, Galangin and kaempferide are different from groups f, g, i, j, k, l and m, but not from each other. i, The 4′-benzoyl flavonol is different from all other compounds except for 4′-methyl, 3′-iodo, 3′-benzoyl and 3′-phenyl flavonol. j, The 4′-methyl flavonol is the same as all other compounds except for quercetin, kaempferol, galangin, kaempferide and 4′-phenyl flavonol. k, The 3′-iodo, 3′-benzoyl and 3′-phenyl flavonol are the same as all other compounds except for quercetin, kaempferol, galangin and kaempferide. l, The 4′-iodo flavonol is different from all compounds except 4′-methyl, 3′-iodo, 3′-benzoyl, 3′-phenyl and 4′-phenyl flavonol. m, The 4′-phenyl flavonol is different from all other compounds except for 3′-iodo, 3′-benzoyl, 3′-phenyl and 4′-iodo flavonol.

**Table III:** Difference between EC_100_ and EC_50_ values in DU-145 and PC-3.

Flavonol	EC_100_ - EC_50_ values in DU-145 cells	Flavonol	EC_100_ - EC_50_ values in PC-3 cells
Quercetin	46.31±8.55^a^	Quercetin	44.26±4.29^h^
Kaempferol	36.01±2.02^b^	Galangin (H-flavonol 5f)	26.81±5.31^i^
4′-Benzoyl flavonol (5b)	24.43±1.10^c^	Kaempferol	24.33±4.63^i^
Galangin (H-flavonol 5f)	20.48±0.46^d^	Kaempferide	19.28±2.10^j^
Kaempferide	19.37±2.02^d^	4′-Benzoyl flavonol (5b)	12.34±0.99^k^
3′-Phenyl flavonol (8c)	11.67±1.11^e^	4′-Methyl flavonol (5e)	10.49±3.29^k^
3′-Iodo flavonol (5c)	10.08±0.84^f^	3′-Iodo flavonol (5c)	9.00±1.06^l^
4′-Methyl flavonol (5e)	9.45±1.89^f^	3′-Benzoyl flavonol (5a)	8.55±2.06^l^
4′-Phenyl flavonol (8d)	6.86±0.18^f^	4′-Iodo flavonol (5d)	6.21±0.59^l^
3′-Benzoyl flavonol (5a)	5.62±5.50^f^	3′-Phenyl flavonol (8c)	4.98±1.54^l^
4′-Iodo flavonol (5d)	2.33±2.05^g^	4′-Phenyl flavonol (8d)	3.35±0.93^m^

EC_100_ - EC_50_ values (μM) are mean ± SEM of 3 independent experiments except for 4′-methyl flavonol in PC-3 cells in which the mean reflects 4 independent experiments. For all flavonols P<0.05. Tukey’s multiple comparison tests determined statistical differences between the EC_100_ values for each flavonol after ANOVA. *DU-145*: a, Quercetin is different from all other compounds. b, Kaempferol is different from all other compounds. c, The 4′-benzoyl flavonol is different from all other compounds except for galangin and kaempferide. d, Galangin and kaempferide are different from all other compounds except for 4′-benzoyl and 3′-phenyl flavonol as well as each other. e, The 3′-phenyl flavonol is the same as all other compounds except for quercetin, kaempferol, 4′-benzoyl and 4′-iodo flavonol. f, The 3′-iodo, 4′-methyl, 4′-phenyl and 3′-benzoyl flavonol are different from all other compounds except 3′-phenyl and 4′-iodo flavonol as well as each other. g, The 4′-iodo flavonol is different from all compounds except 3′-iodo, 4′-methyl, 4′-phenyl and 3′-benzoyl flavonol. *PC-3*: h, Quercetin is different from all other compounds. i, Galangin and kaempferol are different from all other compounds except for kaempferide and each other. j, Kaempferide is different from all other compounds except for galangin, kaempferol, 4′-benzoyl and 4′-methyl flavonol. k, The 4′-benzoyl and 4′-methyl flavonol are the same as all other compounds except for quercetin, galangin, kaempferol and 4′-phenyl flavonol. l, The 3′-phenyl, 3′-benzoyl, 3′-iodo and 4′-iodo flavonol are the same as all other compounds except for quercetin, galangin, kaempferol and kaempferide. m, The 4′-phenyl is different from all other compounds except for 3′-benzoyl, 3′-phenyl, 3′-iodo and 4′-iodo flavonol.

**Table IV:** Viability of HIFF cells treated with flavonols.

A, Viability of cells treated with 50 μM flavonol			

Flavonol	N	% Viability of HIFF (50 μM)^a^	
Quercetin	3	84.47±2.21%	
Kaempferol	3	73.82±1.38%	
Kaempferide	3	52.17±1.17%	
Galangin (5f)	3	47.95±1.38%	
3′-Iodo flavonol (5c)	4	46.79±2.48%	
4′-Iodo flavonol (5d)	3	34.77±0.99%	
4′-Methyl flavonol (5e)	3	37.50±2.27%	
3′-Benzoyl flavonol (5a)	3	34.80±0.20%	
3′-Phenyl flavonol (8c)	3	34.63±2.74%	
4′-Phenyl flavonol (8d)	5	34.42±3.67%	
4′-Benzoyl flavonol (5b)	3	32.33±2.92%	

B, Viability at the average combined EC_50_ concentration of DU-145 and PC-3 for each flavonol			

Flavonol	N	Flavonol (μM)	% Viability of HIFF^a^

4′-Benzoyl flavonol (5b)	3	13	110.92±3.20%
3′-Iodo flavonol (5c)	4	13	105.98±5.79%
Kaempferol	4	36	105.57±5.66%
Quercetin	6	36	94.01±7.67%
Galangin (5f)	4	20	92.63±5.58%
3′-Benzoyl flavonol (5a)	6	12	90.36±4.85%
Kaempferide	3	17	83.91±1.31%
4′-Phenyl flavonol (8d)	4	6	82.09±5.48%
4′-Iodo flavonol (5d)	3	6	70.21±2.70%
4′-Methyl flavonol (5e)	3	12	65.00±4.12%
3′-Phenyl flavonol (8c)	3	6	53.08±9.54%

a Values are mean ± SEM.
